# Reminiscences of an Old Doctor*Read by Dr. Fort (age 77) before the North Texas Medical Association at the special request of the Association.

**Published:** 1905-03

**Authors:** J. M. Fort

**Affiliations:** Paris, Texas


For Texas Medical Journal.

### Reminiscences of an Old Doctor.*

BY J. M. FORT, M. D., PARIS, TEXAS.

Fifty-three years ago I launched my professional canoe out into the turbid stream upon which floats the bark of every general practitioner of medicine.

The system of teaching medicine at that time was not as it is today. All the instruction the medical student had for the acquisition of a medical education was obtained by listening to a series of didactic lectures. As for clinical instruction and bedside experience, that was left for him to obtain at the bedside of his patients in after life.

The young man, fresh from a medical college, who set himself up to administer to the sick and afflicted was nothing more or less, practically speaking, than an educated donkey. It is true he had been taught that all manner of diseases (except the exanthematous) were produced or set up by accidental causes, such as exposure to the various vicissitudes of weather, imprudent diet, etc. The germ-origin of diseases had never been dreamed of.

The opinions then entertained by the great mass of regular physicians were the same as those set forth by Galen in the third century of the Christian era. It should be borne in mind that all progress in medical science had been beaten down and trodden under foot by Ecclesiasticism, which cursed the world and deluged it in blood for so many long and dark centuries.

We were taught, and all or nearly all medical men embraced the opinion, that all febrile diseases, especially those of an acute character, were the result of some localized inflammatory lesion of some one or more of the vital organs of the system; I repeat: this opinion was entertained by the great mass of physicians down to the middle of the last century.

Nervous diseases, except in such cases as hysteria, chorea, epi-

---

*Read by Dr. Fort (age 77) before the North Texas Medical Association at the special request of the Association.

lepsy, etc., were almost lost sight of, both in the diagnosis and treatment of diseases. Even nervous complications were but little regarded by these older physicians.

I well remember we old doctors were very proud of our title. To be called "Doctor" meant something. It was not then an empty word. I remember I would give a negro a quarter to meet me in front of a country church and call me "Doctor" and hitch my horse. I don't think we were to blame for this little bit of egotism, however, for it was before the day when the Osteopath, the Christian Scientist, the Magnetic Healer and the Nigger were made into "doctors." It was before the day when this grand and noble science that taxes the brightest and best intellects of the age to master, if they ever do; this deep and intricate science which shines forth perhaps the brightest star in the galaxy of stars, representing the arts and sciences known to mankind was so debased.

I shall class myself among those veterans and feel proud of the association and say, at that day and time, we seldom tried to diagnose a disease to the extent of naming it. We were governed in the main by the symptoms which presented themselves in each individual case. This nosology or naming of diseases came some time later. If the patient had a high fever, which we were left to judge of by the touch and rate of pulse; we had no such thing as a thermometer. We would bleed the patient to the verge of syncope. If suffering from an acute pain in an accessible locality, we would scarify and cup them or put a fly-blister, something less than a yard square, over the seat of pain and give ten or fifteen grains of calomel; if a child, from three to five grains. As a rule, this sufficed for the first visit to the patient. If the patient died before the next visit, which was usually made in from twelve to twenty-four hours, it was our professional duty to comfort the bereaved family by laying the blame upon the Lord, just as all the preachers do at the present day. We would say to the distressed family and friends, using a pathetic, ministerial tone of voice, "The ways of the Lord are dark, mysterious and incomprehensible. The Lord giveth and the Lord taketh away. Blessed be the name of the Lord." But if the patient luckily survived this treatment but still had fever and seemed quite ill, he was drenched with half a glassful of castor oil and from three to five grains of calomel, alternating with decoc-cinchon or quinine, given from four to six hours. In acute non-febrile cases we depended on emetics and purgatives.

In other respects the case was treated upon the old plan. The patient was closely wrapped in bed—this to promote perspiration. He was put upon a sparing if not a rigid diet and allowed hot drinks, such as sage tea, ginger tea, etc. You must remember that for centuries disease was thought to be an entity, and was gotten rid of by exorcism. Something of this idea must have been entertained by the older doctors in the efforts they made to starve the thing to death. His room must not be ventilated, nor must the patient be allowed cold drinks, not even cold water. Such is a general outline of the practice pursued in those days, the practice being largely routine. It may be reasonably supposed by such large doses and the persistent use of calomel, many of our patients got up from a sick bed with sore mouths. However, the doctor consoled himself by the thought that he had mastered the disease, let it be what it might.

Now don't throw the blame for what you will denominate malpractice upon the shoulders of the older physicians, for they were standing in the front ranks in the profession in their day. Thus far it had progressed and no farther, and, gentlemen, will you dare say they did harm rather than good to suffering humanity? As they were frequently far away from the reach of any aid by a brother physician, alone in the isolated cabin of a pioneer settler, they had to fight single-handed and often successfully the battle of life and death and meet emergencies with a heroism and an undaunted courage which seldom fall to the lot of the physician of today.

At that time tartar emetic was a favorite remedy in the treatment of pulmonary diseases, especially pneumonia after thorough depletion by venesection. Did we or did we not hasten many of our patients out of the world by this course of treatment?

Our armamentarium in the way of remedies consisted, in the main, as follows: Several preparations of mercury, cinchona bark for a time, and then quinine, ipecac, rheubarb, epsom salts, castor oil, tartarized antimony, lobelia, boneset, opium and some of its compounds and a few anti-spasmodics. Add to this a mustang pony and a lariat with which to stake him out.

The main difficulty connected with the intelligent practice of medicine at that time, as it now appears, was the want of knowledge of the true etiology of diseases, and in addition to this, an absence of any conception of the physical warfare which is said to be constantly waged between the disease-producing germs or their ptomaines and the normal vital cell force whose tendency is to resist the invasion of these foreign elements in the system. Here lay what is said to be our greatest mistake, as modern advance and progress in medical science seem to indicate.

Believing, as we were taught, that all diseases were of an inflammatory character, we exerted ourselves to subdue this morbid process, not dreaming, as now contended, that we were robbing the patient of that cell vitality or cell life the innate powers of which constituted his best means of overcoming or combating diseased conditions. And yet, when we consider the success which attended the practice of those older physicians, we must find a then unknown virtue in their remedies, or we are led to doubt the accuracy of the theories now entertained in regard to the origin of diseases.

The cry of the profession now is bacteria—bacteria—bacteria—ptomaines — ptomaines — micro-organisms—cocci—cocci. I think we have gotten down to the root of the tree. We claim that the great majority of diseases are of germ origin. And may not these old doctors have cured many of their cases by the free use of mercury—one of your best germicides?

Individually, I shall never be satisfied with the contention of the germ origin of diseases, as now advocated, until we are shown the origin or nidus of the germs themselves. It is said they are floating in the atmosphere, but the atmosphere does not generate them. You fail to tell us from whence they come, and to my mind this should be the great question which ought to engage the investigating minds of the physicians of the day.

There was another circumstance which, in my judgment, tended to the success of the practice pursued in those days, namely, this whole southern country was being converted into an agricultural country. Lands were being cleared up and thousands of acres of virgin soil were being exposed to the rays of the sun. Consequently malarial fevers and malarial complications of diseases prevailed to a very great extent throughout the entire southern country. This contention, as I am aware, presupposes malaria to be a product of the soil which I am thoroughly convinced is true, the mosquito theory to the contrary notwithstanding.

This modification or complication of diseased conditions may have had, and doubtless did have, its influence in their favor. Here we at least find indications for the use of mercury and quinine. Believing, as we did, and as we were taught, that all fevers were the result of an inflammatory process, as before stated, we exerted ourselves to subdue this process, not realizing, as is now contended, that we were robbing the patient of his constitutional and reserved physiological powers of resistance, the powers of resistance now relied upon to carry our patients safely through a disease.

To illustrate the importance attached to venesection as a remedy in the treatment of diseases at that time, let me say that even septic fevers were regarded as inflammatory affections, and it was the sworn duty of the physician to bleed such cases first, last and all the time. Sepsis, as now understood, was an unknown condition. We saw puerperal women lie in this condition, but never dreamed that it was the result of absorption of a poison through local abrasions, or retained debris in the uterine cavity. That illustrious teacher of obstetrics, Chas. D. Meigs, was in the habit of detaching, in imagination, the peritoneal membrane and spreading it out before his class and telling us that puerperal fever was an inflammation of this broad sheet of membrane, and that nothing short of the lancet could rescue the patient from immediate and certain death. He went so far as to say, "Don't wait to fix a binder with which to cord the arm, but snatch the garter from the leg of some old woman, always present, and set the stream to flowing." In those days the great majority of obstetric cases were bled from once to twice before the time of parturition and, as a result, I think we had fewer cases of eclampsia then than now. This, however, is but an opinion.

The first obstetric case which I attended after beginning the practice, was a lady in one of our nicest families, a primipara, a handsome and lovely lady with all her environments in keeping with the personnel of the family. The old lady, mother of my patient, was on hand, of course. The old lady looked at the young, dudish-looking doctor out of the corners of her eyes with an expression which admitted of several interpretations. I put on my most knowing looks and all the professional airs I could command. I realized at once the importance of gaining the confidence of this old lady. Otherwise, I would be a gone chicken. So, as soon as it was convenient, I took a seat by her side and proceeded to make all manner of inquiries about the health and general constitutional condition of her daughter from the beginning of her pregnancy up to the present time. After this I took a seat beside the bed and made a digital examination of the case. And what did I learn from the examination? I knew no more than if I had introduced my finger into a tub of guts. I didn't know the os uteri when I touched it. I felt around the bony cavity, and satisfied myself that there was room for the head of an ordinary child to pass without bursting things to pieces. So I determined to let nature take its course, a wise conclusion in more instances than this one. When the pains became severe and bearing down, the poor woman looked to be in such distress and cried out so lustily, I got scared. I really thought the woman would die. I had never seen a woman in labor before. I looked at the old lady, her mother, and saw that she was not at all alarmed. She was taking things easy. This reassured me, and I became reconciled to my fate. The labor proceeded normally, and when the baby came into the world, I was ready to catch it. I did with the baby and also with my patient everything suggested by the old lady, and it all ended well, and "All's well that ends well." But if it had been a case of placenta previa or eclampsia, I am sure I would have died as well as the patient, for I was as ignorant of the ins and outs of even the physiological process of labor as a cat of holidays.

All eruptive fevers were then believed to have their origin in, and to be propagated by an unknown, intangible effluvia, or poisoned atmosphere. The question as to what this disease-producing poison was, was never considered. It was taken for granted just as thousands of erroneous conceptions are taken as facts at the present day. Some old, forgotten smart Alec said it was so, therefore it had to be so. All these cases (i. e., eruptive fevers) were treated by close confinement in a warm room, the giving of warm drinks and sweating teas. Free ventilation, cold water or cold drinks of every kind, and cold sponging were strictly prohibited in all cases. Had a physician pursued this,—what we now call a rational course of treatment,—in any given case, at that time, and from some unforeseen complication had lost the case, you might as well have said "Farewell, brother Crawford, ah," to that doctor. He could have done no more practice in that community. Doctors and laymen alike would have denounced the treatment and hounded him as a reckless humbug.

For several years after I began the practice of medicine, all the older practitioners said that, up to that time, there had never been a case of typhoid fever west of the Mississippi river. Whether this opinion was entertained from the fact that no such cases had occurred prior to that date, or whether it was in consequence of their inability to diagnose these cases, I am unable to say. I do know, however, that I had not been engaged in the practice very long before I ran across cases of this disease, and I did, as is frequently done now, kill some of them trying to cure them with medicines. The great mistake made in the treatment of this disease, in my judgment, is too much medication and not enough attention to the person of the patient.

We did but little surgery in those days. As a rule, the doctors were as timid in the use of the knife as the patients were in having it used. It is true, we had an arm or a leg to amputate from necessity now and then, and occasionally to splint a fractured bone. I remember assisting my father when a boy in amputating an old fellow's leg for necrosis of the tibia. We extemporized an amputating table out in the yard. It was before the days of anæsthetics, so we tied the old fellow down hard and fast with a rope. He yelled manfully during the operation. Being a mischievous boy, it was fun for me. After his leg was taken off, one of his boys asked the old man what he should do with it. He said, "Throw the damn thing to the dogs."

We knew nothing of ovarian cysts or fibroid tumor or appendicitis. To perform laporotomy would have been a crime never to be forgiven. Now and then some poor, innocent girl would go down to her grave in shame and disgrace on account of the ignorance of the profession in regard to her malady.

For more than a thousand years Ecclesiasticism, a system of organized tyranny, put its heel of oppression upon the science of medicine, and, in fact, upon all science, denied to all men the right of individual opinion, tying them down to the hypocritical pretense of a uniform belief in the dogmas and dicta of its corrupt organization, denied to the profession all right of dissection, "saying that the lifeless body of an individual should not be cut, as it would mar and deface it when resurrected from the dead on the judgment day," that imaginary day of sham and pretense. Ah, who can estimate the tears that have been made to flow, how mankind has been made to suffer, how tortured, how the hearts of even poor, innocent women and sweet children have been torn asunder and made to bleed. What a cry of wail and woe has ascended and fallen upon the ears of a just and merciful God by this ecclesiastical fraud, this unprincipled and dishonest mode of distorting money from the poor and illiterate masses.

The physicians of that time were very different from some of our young doctors of today. In some instances the first thing a young doctor endeavors to do now is to establish a reputation of being a first-class surgeon by cutting and slashing every poor woman who falls in his way, often succeeding better in filling up graveyards than in establishing an envied reputation. I like to see an ambitious, aspiring young doctor rise in the profession, but I have no patience with or respect for the man who would sacrifice human life recklessly for the sake of an ephemeral reputation, a reputation that would go down with the setting sun.

Then again, allow me to say that all the modern advances in the way of appliances as aids to the diagnosis of diseases were unknown to the older doctors. They had not been reached in the onward progress of the science. Even medical nomenclature is getting beyond the reach of some of them. They were in the main a fearless set of men, daring to do their professional duty under the most adverse and unfavorable circumstances. It was never too hot or too cold; it was never too wet or too dry; never too dark or stormy for these old veterans of by-gone days to listen to the cry of the afflicted or to dry the tears of the distressed.

Let me beg you, gentlemen, to lay the hand of censure lightly upon their shoulders; suppress the gesture of scorn, and silence the word of ridicule offered these old doctors. They did as one of old. They did the best they could. When a slur is about to be cast upon them, remember that medical science has made its rapid strides in progress since their day. They lived and labored in the twilight of demonstrated medical science, and now they have gone to their reward. "Peace be to their ashes," and respect for their memories.

